# Supporting parents by combatting social inequalities in health: a realist evaluation

**DOI:** 10.1186/s12889-021-11237-2

**Published:** 2021-06-29

**Authors:** Annabelle Pierron, Laurence Fond-Harmant, François Alla

**Affiliations:** 1 Bordeaux Population Health Research Center, UMR 1219, Méthodes pour la recherche interventionnelle en santé des populations, Université de Bordeaux, 33000 , 1 rue Jean Burguet, Bordeaux, France; 2 Directrice de Recherche. ACSAL Agence pour la Coopération Scientifique, LEPS UR 3412-Université Sorbonne Paris Nord, Afrique, Luxembourg; 3 Bordeaux Population Health Research Center, CHU INSERM, UMR 1219 CIC-EC 1401, Université de Bordeaux, Bordeaux, France

**Keywords:** Social determinants of health, Parenting, Health status disparities, Intervention research, Theory, Complexity

## Abstract

**Background:**

To reduce social inequities in health, the World Health Organization’s Commission on Social Determinants of Health recommends acting as soon as life begins. In this context, parenting support is promoted as a major lever.

The objective of the present research was to develop an intervention theory establishing the conditions for the success of interventions, policies, and organizations supporting parenting in terms of reducing or preventing social inequalities in health for both mother and child in the perinatal period.

**Methods:**

To meet these objectives, we conducted a realist evaluation based on a multiple-case study. The study evaluated two border towns in Europe. We collected data from three sources: documentary reviews, focus groups and interviews with professionals, and parental questionnaires.

**Results:**

The main results concerning the fight against social inequalities in health show a true willingness on the part of those involved to carry out universal actions, coordinated between professionals and institutions, in response to the demands of parents; however, the reality on the ground shows the complexity of their implementation and the multiplicity of results. Our middle-range theory showed that to be effective in tackling social inequalities in health, actions must address structural determinants at the macro-systemic level. However, the field of realist evaluation shows that it is first and foremost the actions focused on individual behavior that are implemented.

While there is a general political desire to combat social inequalities in health in early childhood, the results show that the strategies in place are potentially not the most effective. Effective support actions would respond to individual strategies; however, current approaches target parents’ behavior, aiming to empower them but without giving them the means to do so.

**Conclusions:**

This research constitutes a body of knowledge gathered for reflection and action. In particular, any perinatal policy should clearly state among its objectives the intention to reduce social inequalities in health. The policy should also state that it will be evaluated according to the criteria of proportionate universalism, interprofessional coordination, and actions based on the diversity of parents’ needs.

**Supplementary Information:**

The online version contains supplementary material available at 10.1186/s12889-021-11237-2.

## Background

Social inequalities in health are defined as significant and unfair health differences between social groups or geographic areas. These differences are due to the conditions in which people are born, live, grow up, learn, work, and age. To reduce these inequities, the World Health Organization’s Commission on Social Determinants of Health recommends acting as soon as life begins. In this context, parenting support is promoted as a major lever [1].

Health promotion interventions, actions, and programs relating to parenting support have been the subject of many publications showing their efficacy in improving parental and newborn health. We recently conducted a synthesis of literature reviews on effective parenting support interventions in the perinatal period and how they contribute to reducing social inequalities in health [1]. That review showed that low-cost interventions offer significant benefits, particularly by increasing the self-esteem of mothers, reducing their anxiety and stress, and improving infant sleep. The most effective actions are those that begin during pregnancy and in which parents actively participate. Our work also showed that, typically, the authors of the reviews did not explicitly consider social inequalities in health. Few authors have addressed the notion of health equity, and their vision of social inequalities in health has remained limited. In particular, parenting programs, most often offered to mothers, and especially the most disadvantaged, rarely take social gradients of health into account [1].

Our synthesis revealed the limits of current knowledge on health equity in the field of parenting support. Moreover, scientific papers do not generally contain much detail on the conditions of implementation of interventions, their contexts, processes, and mechanisms [2–6]. It seems essential, therefore, to undertake field research to study how inequalities are considered in interventions and organizations that support parenting.

Parenting interventions are generally complex and highly dependent on the context in which they take place. Evaluating them with regard to social inequalities in health requires an understanding of how and for whom they work, and under what conditions. An evaluation based on theory makes it possible to address this challenge [7–11].

The objective of the present research was to develop an intervention theory [8–10] establishing the conditions for the success of interventions, policies, and organizations supporting parenting in terms of reducing or preventing social inequalities in health for both mother and child in the perinatal period. To meet these objectives, we conducted a realist evaluation [12–14] based on a multiple-case study.

Such an intervention theory would make it possible to propose professional recommendations using contextualized intervention levers.

## Methods

The presentation of our methods and results follows the RAMESES II reporting guidelines [15].

### Conceptual and methodological framework of the research approach

Parenting behaviors are strongly dependent on the sociocultural context and on health and education policies. Parents’ behaviors are constantly evolving; parents gradually adapt as they interact with their newborn child, family, and environment. Parenthood is a unique experience for each person, affected by cultural and social expectations and lifestyles. Support offered to pregnant women, mothers, and parents during the first weeks of their child’s life can take a variety of forms, whether for groups or individuals, and among target populations or more universally. Many different stakeholders are involved in this process: health professionals from various disciplines such as obstetrics, midwifery, pediatrics, general medicine, and psychology, and professionals from the social sector such as education and community organizations (educators, social workers, etc.). These stakeholders interact with each other, parents, and institutions. Such interactions can take multiple forms, from a single seminar to the development of networks, and from multidisciplinary meetings to informal discussions. Because of this, and because parenting is not just the individual responsibility of parents [5, 16], parenting support interventions present the characteristics of complex interventions as defined by the Medical Research Council (MRC): “[complex interventions are those] comprising several interacting components, though the complexity of their implementation may add additional complexity, as does the number of organizational levels targeted” [8].

Evaluating health promotion interventions in the field of parenting support, particularly with the aim of reducing social inequalities in health, requires a systemic and dynamic approach in order to address their complexity [7, 11, 17]. As Moore et al. argue, the “complex systems perspective broadens the parameters of ‘relevant’ evidence and theory” [9]. The MRC has developed a theoretical framework to evaluate complex interventions in order to understand how, for whom, and under what circumstances an intervention is effective or not. In this framework, the context interacts at all levels of the intervention: from the initial hypothesis to its implementation, impact mechanisms, and outcomes. These interactions function as feedback loops: the intervention modifies the context, which itself modifies the intervention. This framework exists within a social dynamic that must be considered when theorizing about the ongoing functioning of interventions. Once theories about the dynamics of the system have been generated, hypotheses about action levers can be formulated [12, 18–20].

To clarify the hypotheses of causality, the MRC recommends describing the intervention in a logic model and using the systems approach as a key to understand the complexity of situations. We adapted the socio-ecological model from Bronfenbrenner’s nested systems theory to our research question. We present our adapted model in our supplementary material file 1. Bronfenbrenner [21, 22] developed the idea that behavior is a function of interactions between people and their environment.

This systems model is particularly useful for evaluating health promotion interventions. Absil showed how Bronfenbrenner’s nested systems model is “well-suited to modeling a determinants of health approach” [23, 24]. When individual behavior is viewed as resulting from multiple interactions between the individual and their surrounding and limiting environment, the health care system itself can be seen as an action lever for health promotion.

The logic model presents systemic elements but does not explain the mechanisms through which these elements interact with each other. For that, we need a theory. “The theory is the interpretative framework for defining strategies, their implementation, the expected outcomes and all the investigation methods” [25]; generating a theory of intervention therefore requires investigation, and field research serves to validate the hypotheses we formulate using data from the literature. We chose realist evaluation as the method to do this [14].

Potvin shows how modeling the functioning of complex interventions requires “understanding how a program and its context transform each other” [7, 11, 26]. To do so, it is necessary to explore the complexity of the actual context; that is, to conduct field research at the local level. Several methods can be used to determine the systems in place and how they interact. In the field of health promotion, a lot of current research uses mixed methods that are both qualitative and quantitative to describe the context, mechanisms at work, and outcomes that result from the interventions [13, 27–29].

Evaluators are interested in the change mechanisms related to human reasoning [30, 31], which are theoretically neither controllable nor linear (since they strongly depend on context). Theory-driven evaluation, including realist evaluation [10, 32, 33], could take these dimensions into account.

Moreover, understanding whether an intervention works is important but not sufficient: Pawson and Tilley [14, 18] developed a methodological approach called realist evaluation to address the lack of understanding of how interventions function. Realist evaluation allows us to understand and theorize “how complex interventions function and in which circumstances” [34–36]. The idea is to investigate the stakeholders and populations involved as close as possible to where the interventions take place in order to explain how they function. Generating theories about how interventions function involves offering hypotheses based on existing data, then comparing these hypotheses to empirical data collected in the field. Several entities are formed linking context to effects, explained by mechanisms, within a structure that Pawson and Tilley call a context–mechanism–outcome configuration. To improve understanding, the theory is further refined using an iterative process whereby the theories are revisited and the empirical data are then analyzed [14].

New, readjusted hypotheses called middle-range theories are then developed. They address the context and can explain how the intervention affects change and vice versa. The theory is enriched by comparison with different contexts via this process of iteration, in which several case studies are conducted. From intermediate theory to intermediate theory, regularities in the relationship between the context, mechanism, and effects (or outcomes) of the intervention appear [19, 29, 37].

### The theory of intervention: initial hypotheses

Our realist evaluation began with a synthesis of the hypotheses about how these interventions function, based on data from literature reviews. The initial theoretical foundations of our research were as follows [1].

A maternal health promotion program in the perinatal period that promotes parenting support will be effective in the fight against social inequalities in health if it:

- begins at the start of pregnancy and continues into the postnatal period [38],
- has diverse approaches, taking into account the organization of neighborhoods, cultural and social expectations, and lifestyles [5, 16, 39],
- is organized according to proportionate universalism, which takes into consideration all social and economic layers of the population [38, 40],
- considers parenting broadly rather than focusing uniquely on maternal behavior [2, 4, 5, 16, 41], and
- is co-developed as part of a shared approach between professionals and institutions, based on the needs of families rather than professional standards [2, 4, 40, 42].

Our recent literature review identified certain levers present in parenting support interventions that were described as effective in reducing social inequalities in health [1]:

- Proportionate universalism refers to universal measures (destined for all parents) that are proportional to the needs and obstacles that certain populations face (adapted to certain target groups). This presupposes a recognition of the importance of not stigmatizing certain groups and using diverse modes of action. For example, it could take the form of informal meetings between parents in the most economically disadvantaged neighborhoods (perhaps in a toy library, a literary gathering cafe, or a green space). It could also involve adapting modes of support to the expectations of parents, either in institutions or at parents’ homes.
- The coordination of facilitators involved in parenting support refers to practices that ensure continuity and complementarity in the support provided (in terms of a logical trajectory). This presupposes that professionals understand the institutions, measures, and modes of support provided in the area. For example, programs or practices that begin during pregnancy could be coordinated and continue into the postnatal period, involving professionals from various disciplines and institutions.
- The integration of the difficulties of daily life refers to the mechanism through which programs and practices for mothers and fathers are known to them, accessible, adapted to their needs, and consider extended family, such as grandparents, brothers and sisters, and friends, as a potential resource. This mechanism presupposes the consideration of the living environment in terms of the material or organizational limitations parents face, particularly access to childcare options in their neighborhoods.
- Active parent participation refers to the ability of parents to participate in the development of programs and interventions designed for them. This presupposes that professionals and politicians are aware of issues relating to health literacy and the concepts of empowerment and democracy in health, including issues relating to urban planning and the geographical organization of health services.

**Fig. 1:**
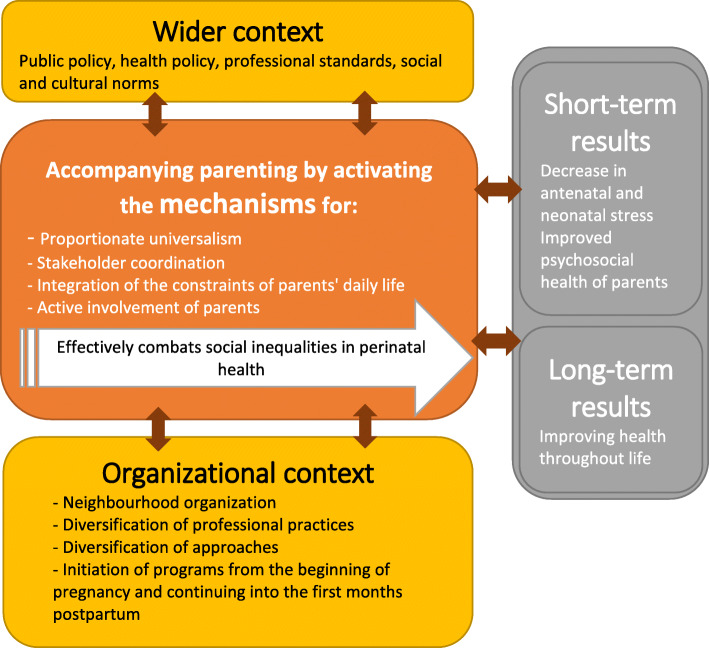
Initial theory of combatting social inequalities in health via support for parenthood in the perinatal period

Our initial theory is presented in Fig. 1.

### Design

**Fig. 2:**
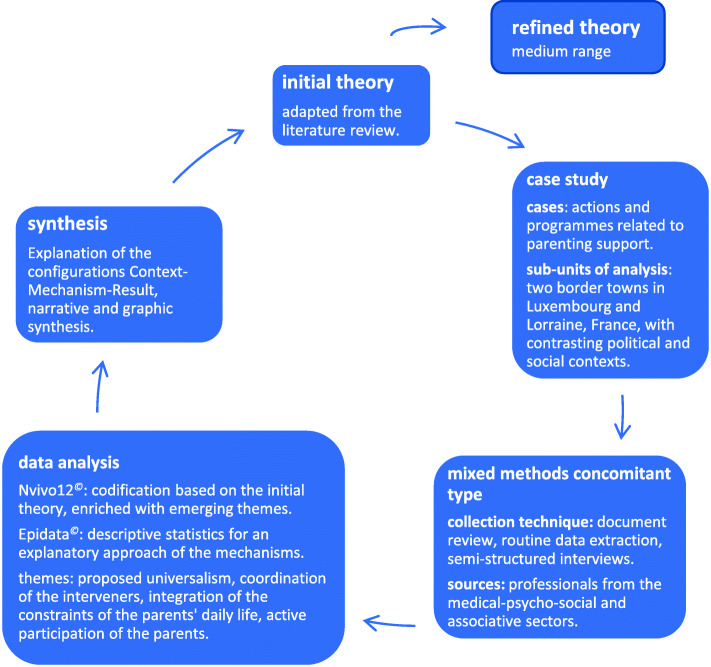
Realist evaluation cycle

This study is a realist evaluation based on a multiple-case study. The realist evaluation cycle is summarized in Fig. 2.

### Population

The study evaluated two border towns in Europe: Esch-sur-Alzette in the Grand Duchy of Luxembourg and Longwy in France. They were selected because of their geographic proximity and the particularly vulnerable and inequitable conditions facing the populations who live there. The advantage of this pair of towns is that they border each other, yet face different social, political, and institutional realities.

The closure of iron mines affected the steel industry in both towns, and people in this area face high rates of unemployment. The attractiveness of Luxembourg’s labor market has prompted increased movement of workers across the border in the last 10 years or so. Although this new economic upsurge may seem positive, there are significant differences in lifestyle between native people and foreigners [43–47].

### Data collection

We collected data from three sources: documentary reviews, focus groups and interviews with professionals, and parental questionnaires.

#### Documentary reviews

Documents such as the websites of community organizations or maternity wards and flyers for the general public helped us understand the purpose of the intervention as presented in the document: who offered what, how, for whom, in which circumstances, with what kinds of collaborations, for what expected results, especially in terms of reducing social inequalities in health, and whether these interventions are universal, targeted, or gradual.

Moreover, we consulted statistical data published on governmental websites (Institut National de la Statistique et des Etudes Economiques [INSEE], Direction de la Recherche des Etudes de l’Evaluation et des Statistiques [DREES], and institut national de la STATistique et des études EConomiques [STATEC]) to determine the demographic context, employment conditions, and standard of living in both areas. Perinatal health data for these areas came from public health databanks (Agence Technique de l’Information sur l’Hospitalisation [ATIH], Euro-Peristat, and STATEC). This step allowed us to understand the general context in which public policies, programs, and actions are set. The search for indicators at the scale of the two towns made it possible to specify the local socio-demographic profiles.

#### Focus groups and interviews

We organized discussion groups with professionals in the field. We wrote an interview guide and established the focus groups. Our reporting complied with the Consolidated Criteria for Reporting Qualitative Research recommendations [48] according to the three areas of control: research team and reflexivity, study design, and data analysis and results.

Healthcare and medical-social professionals working either independently or in hospitals, medical-social centers, or house visits were included in this study on a voluntary basis. We asked pediatricians, obstetricians, general practitioners, midwives, pediatric nurses, psychologists, social workers, and those working with community organizations involved in parenting support to share their thoughts with us.

Starting with levers that were viewed as effective in fighting social inequalities in health, as described in the literature review [1], we examined practices through the words of these professionals. We asked them to react to our presentation of the results from the quantitative and documentary study. The focus groups had a twofold objective: first, in group interviews with those working in the field, to understand their individual practices and whether they were involved in interdisciplinary and multidisciplinary collaborations and, second, to allow these same participants to understand their own ideas about social inequalities in health by informing them of the results from the documentary and quantitative analysis. We also held individual interviews with parents to validate, confirm, refine, and guide our hypotheses.

Semi-directed individual and group interviews were conducted from May to August 2019. Regulatory and financial obligations required a more modest deployment of the survey than envisaged in the initial protocol. We conducted seven individual interviews and met with 25 professionals during focus groups. The diversity of profiles of the field actors was a priority of the survey in order to explore multiple points of view. The in-depth interviews with professionals from the health sector allowed us to explore their social representations and their practices in relation to support for parents. We conducted interviews with two pediatricians, four midwives, and one psychologist. We conducted three group discussions with five social workers, two doctors, three midwives, eight childcare workers, one educator, one occupational therapist, two psychologists, and three secretaries. We visited two associated organizations, in the context of an open discussion. The interviews conducted with these field actors served to identify the context in which the actions and interventions were carried out in terms of strengths, weaknesses, opportunities, and threats (a SWOT strategic analysis grid).

We recorded our discussions during the focus groups and interviews. The verbatim reports were transcribed and processed using NVivo® 12 software. We then analyzed the content and conducted a multidisciplinary analysis of the overarching themes. We made notes on our observations and incorporated them into the analysis.

The focus group discussion guide in English language is submitted as supplementary file 2.

#### Parental questionnaires

The questionnaires for parents (*n* = 350) developed for this study were translated into several languages (French, German, Portuguese, English, and Arabic). They allowed us to determine whether they knew about measures and activities designed to help them, had access to these interventions, felt the need for them, and were satisfied with them. The questionnaires allowed us to evaluate three components: the actions and measures in place and each respondent’s personal support network and socioeconomic status. The parental questionnaires also allowed us to analyze whether the parenting support interventions met parents’ needs and expectations, and whether they were satisfied with them. To determine how social inequalities were considered in existing programs, we analyzed this information according to the socioeconomic strata of the respondents. The English language questionnaire is available in supplementary material file 3.

To recruit parents, we brought posters to consultation areas and parenting support organizations (*n* = 15 places). These posters asked parents to participate in the study in several languages: French, German, Portuguese, and Arabic. We collected 125 usable questionnaires in Esch-sur-Alzette and 85 in Longwy. Eight were rejected as invalid due to a lack of key information.

### Analysis

#### Analysis of different materials

For the documentary corpus, we used the REFLEX-ISS tool to conduct a systematic analysis of how social inequalities in health were addressed [49, 50]. This tool is a grid that was developed and evaluated to “adjust and improve a planned or existing intervention at different stages of its life cycle”. For our documentary analysis, we created a grid that we adapted from REFLEX-ISS, taking several themes into account: planning, implementation, and empowerment. We analyzed concepts relating to evaluation and long-term viability whenever the documentary source made this possible. The grid is presented in our supplementary material file 4.

The analysis of verbatim data was carried out according to the recommendations of Bardin [49]. Coding was based on the themes selected when the initial theory was formulated in conjunction with consideration of social inequalities in health. The mechanisms selected beforehand for testing concerned characteristics related to proportionate universalism, coordination of caregivers, integration of the constraints of parents’ daily lives, and active parental participation.

The data from the questionnaires were processed using Epi Data® 3.1 software. We used Epi Info® 7.2.2.6 software for the statistical analysis.

#### Mixed methods

Our design used mixed methods, such as an embedded design in which qualitative and quantitative methods are used simultaneously [36, 37]. Here, the quantitative methods support the qualitative one. In our supplementary material (file 5), we provide a figure that summarizes the interweaving of these approaches, based on the framework of Guével and Pommier [51, 52].

#### Realist analysis

Our analysis was intended to generate hypotheses that explain how and why an intervention is supposed to have an effect [28, 37]. This involves comparing the context–mechanism–outcome configurations with what happens in the field. Ridde and Robert described how to develop these theories in an operational manner: “[n]either inductive [empirical observations that identify regularities that can lead to a theory] nor deductive [theoretical hypotheses that are tested against empirical observation]” [24]. Rather, the idea is to go back and forth constantly between empirical observations and refined theories. The configurations are expressed as probable hypotheses about how the interventions work. The data used to develop these configurations are then adapted to the mechanism being studied. The hypotheses were presented to the parents and professionals to validate, reject, or clarify the research team’s proposals.

All the information from the quantitative and qualitative documentary collections enabled us to understand the interactions between the needs of parents and the services offered by professionals in the context of health policies. To understand how, why, and for whom programs and actions combat social inequalities in health, it was necessary to reveal the interrelationships between context (hereafter, identified in the text with a “C”) and results (“R”).

To establish the interactions between the elements, we proceeded in several steps. Using the NVivo® software, we began by classifying the verbatim extracts according to the mechanisms (“M”) of the initial theory to which they refer. For example, for the “stakeholder coordination” mechanism, we grouped together the elements related to working meetings, the inter-professional exchanges resulting from the verbatim data, and elements from the corpus of documents related to the national public health strategy.

Then, around these groups of verbatim data, the contextual elements that could potentially influence them were sought. To continue with the same example, some contextual elements were “training courses allowing the use of a common language” from the descriptions of professionals’ practices in their verbatim data, or “the perinatal network discusses and edits recommendations” in the documentary corpus.

At this stage of the analysis, we had classified the elements of context and mechanism; results were linked via heuristic maps around key words. These maps were an ideal tool to concretize the links between the elements. Gradually, the interacting elements led us to a better understanding of “who does what for whom and how”. The verbatim data were read several times, revealing certain elements that were sometimes classified as context-related and sometimes as related to the mechanism within the framework of another context–mechanism–result articulation. For example, “I don’t attend meetings” was the result of “I already work a lot and I don’t have time to spend on it” and also an element of context for “I don’t understand how decisions are made”.

Similar lexical fields were revealed by our analysis of the verbatim data, which left some underlying contextual elements that were results or mechanisms that had not initially been considered. For example, the lexical field of institutional constraints emerged.

### Ethics and consent

This research was validated by the National Research Ethics Committee of Luxembourg (reference, CNER 201801/03). The survey was based on the voluntary and anonymous participation of the participants, with each participant being free to withdraw from the study at any time. The consent of the participants was verbal - because inclusion was anonymous and voluntary- as approved by the ethics committee.

## Results

In France, the national strategy plan to support parenthood proposes various lines of action, such as providing home support after parents leave the maternity hospital or raising awareness among professionals about support services for parenthood. In the Grand Duchy of Luxembourg, the health plans in force, which include a section on early childhood, refer to public health advice and recommendations. France and Luxembourg authorize maternity, paternity, and parental leave, the duration and compensation arrangements of which differ between the two countries.^1^ The two territories collect epidemiological data and coordinate their practices in accordance with the recommendations issued by the perinatal network in each region.

The two towns studied have high unemployment rates compared to national data: in 2018, 20.2% in Longwy (vs. 9.1% in France, Institut National de la Statistiques et des Etudes Economiques data) and 10.1% in Esch-sur-Alzette (vs. 5.9% for Luxembourg, STATEC data). The interventions, professionals, methods used, and programs are relatively similar in the two towns. Essentially, the objectives correspond to programs aimed at parental knowledge or maternal behavior. The methods used are multiple: courses, information sessions, consultations, and interviews. The sessions take place individually or collectively, in institutions or at home. The actors are health or education professionals from the voluntary or social sectors. The methods of adaptation with regard to combatting social inequalities in health show that certain actions are proposed in various languages; many of the interventions are free of charge for parents.

### Results based on the initial hypotheses

#### Proportionate universalism

One of the first elements analyzed was the universal nature of the interventions. Many interventions in France (“FR”) and Luxembourg (“LU”) are offered systematically to all parents, most of them free of charge. They are financed by the state from a national or regional budget or reimbursed by health funds (Caisse Nationale de Santé in Luxembourg and Caisse Nationale d’Assurance Maladie in France).

Nearly all births take place in hospitals, so all women who are pregnant or have given birth are invited to interventions offered by the maternity ward. It is also in the maternity wards of the two towns that relevant associations (and territorial institutions in France) present their activities, via flyers or during specific interventions. As soon as they leave the maternity hospital, all young mothers are informed of the possibility of benefiting from home visits. However, the presentation of these universal interventions is not always universal in practice.

##### Language barrier

All parents receive universal information (LU and FR) on the services intended for them (C) via written, spoken, and displayed media provided by various professionals in contexts where the language barrier (C1) and time at which the information is provided (C2) strongly influence the results, depending on the degree of literacy (M1) and ability to understand (M2) or accept (M3) the messages according to the person’s cultural references. The language barrier contributes from the outset to non-access (R) to various services when parents are informed by post; this is particularly the case in Longwy (FR), where a letter is systematically sent informing parents of the availability of maternal and child protection services.

“At birth preparation sessions, only women who speak French are allowed to attend,” said one midwife (AL); birth preparation courses are given in several languages in Esch-sur-Alzette (LU), whereas this is not the case in Longwy (FR). It would appear that language problems are less prevalent in Luxembourg than in France. Historically, Luxembourg has developed a multi-linguistic culture.

Professionals use translation tools or the help of interpreters who are mostly family members. Unanimously, professionals in the two territories studied have described difficulties related to the language barrier. The intervention of a professional interpreter or the family, although frequently used, raises questions about privacy, given that this is required from the outset with regard to parenthood. These linguistic limits also raise issues related to cultural and social references and representations that are difficult to address, even though they are at the heart of the matter.

##### Voluntary versus financial incentive interventions

All parents can benefit from free services (C1), such as immediate postpartum home visits and consultations in social medical centers or health education sessions in associations or maternity wards. The use of services is influenced by their free and optional nature: paying for a consultation encourages the use of certain services as this payment is a guarantee of an assertive, personal approach (M2) or ensures quality care (M1). The cost of obstetrical and pediatric medical consultations must be paid in advance entirely in Esch-sur-Alzette (LU), and partially in Longwy (FR), where attendance is remarkably high (R1). The allocation of family allowances is an incentive to attend (C2) compulsory medical consultations, which are much more frequent (R1) than optional services (R2).

The compulsory or optional nature of visits and consultations has an impact on attendance, causing tension between the different disciplines, as a nursery nurse (CE) testifies: “Between two free services, parents prefer to go to the doctor, because when the doctor says something it is more valuable.” In both Esch-sur-Alzette (LU) and Longwy (FR), to be entitled to certain benefits, parents must prove that they have undergone several consultations which are considered compulsory, as described by this pediatrician (BE): “People come every month, it’s planned, organized like that.”

#### Coordination of those involved in parenting support

Coordination between actors is limited, with different configurations in the two towns. Generally, in Esch-sur-Alzette (LU), actions are not well coordinated with each other (C1). The historical context of professional competition between professionals and community services remains an obstacle (M1) to communication and to the referral of parents to colleagues or support institutions (R1). In Longwy (FR), as part of the organization of pregnancy care and the post-partum return home, in accordance with the national recommendations implemented by the perinatal network (C2), professionals refer parents to structures or workers from other disciplines in a manner typical of similar countries (R2), without the professionals knowing each other or exchanging information.

In the two surveyed locations, access to multidisciplinary training exists (C) and professionals engage in this training to a highly variable extent depending on whether they have legislative or institutional constraints. Arguments concerning the availability of time (M1), cost of training (M2), and financial loss during training time (M3) seem to have a strong impact on the lack of such investment (R1). Conversely, personal motivation linked to an interest in networking (M4) favors involvement in multidisciplinary training (R2).

In terms of the complementarity of interventions, two contexts interact with the results. Both towns organize multidisciplinary and multi-institutional meetings (C), enabling comprehensive and coherent support for situations (R), but almost exclusively in the context of reporting situations (M) where children are potentially in danger or parents are in socioeconomic difficulty. Also, both towns suffer from a lack of pediatricians (C1). It is the lack of confidence (M1) in the skills of other professionals and institutions or the lack of staff (M2) that partly explains the excessive numbers of requests for pediatric consultations in the emergency department in Longwy (FR; R1) and in Esch-sur-Alzette (LU; R2). It should be noted that many of these consultations are not strictly based on a request for medical care, but on offers of support by other professionals or structures who have suggested this to parents (R3).

#### Integration of constraints linked to daily life

Consultations are accessible to parents who benefit from an organization that frees them from constraints and offers them access to consultations via a means of transport. Women who have difficulties attending consultations (R) may be isolated (C1), have other children (C2), speak a foreign language (C3), be unfamiliar with the health system (M1), have difficulties freeing themselves from constraints (M2) or moving around town (M2), or be limited when expressing themselves (M3).

The family environment (C1) represents both an organizational and emotional support (R1) and a constraint (R2) for parents who sometimes feel judged by their relatives (M). Advice given by their family and personal support network (family, friends) raises issues of influence, as described by a pediatrician (PL): “Mothers need to see how other mothers do it, and not feel guilty about not doing what they are told. Grandmothers looked after their babies differently 20−30 years ago.”

#### Active involvement of parents

Some families, particularly the most socially (C1) or economically (C2) deprived, seem to benefit little from the services (R). In the two locations studied, professionals report that parents are afraid to show that they lack knowledge or financial means (M). Thus, home visits are few in number among these families (R1), who prefer consultations in an institution or with a health professional (R2) so as not to reveal their living environment.

Generally speaking, social services are perceived negatively; they are seen as a threat to the freedom to exercise parenting according to one’s cultural references, i.e., differently from the standard set by professionals (M1). Moreover, social services are perceived as presenting a threat of child placement, with a risk that the parents will be reported to the legal authorities (M2), as described by a midwife (ML), “People are not aware of the PMI [social services department] and there is fear of reporting, child placement, intrusion into family privacy.” Additionally, as a social worker (IE) noted, “The people we think are in need, where there really are social or other worries, these people say to themselves that they are already stigmatized and if they go there they will be doubly stigmatized!”

Parents at the highest social and cultural level (C1) show their needs, questions, and doubts (R1), and in consultations seek assurance, self-confidence, and legitimacy for their actions (M1), whereas parents in great social, economic, psychological, or educational difficulty (C2) are afraid (M2) to ask for help and consult little (R2).

### Middle-range theory

**Fig. 3:**
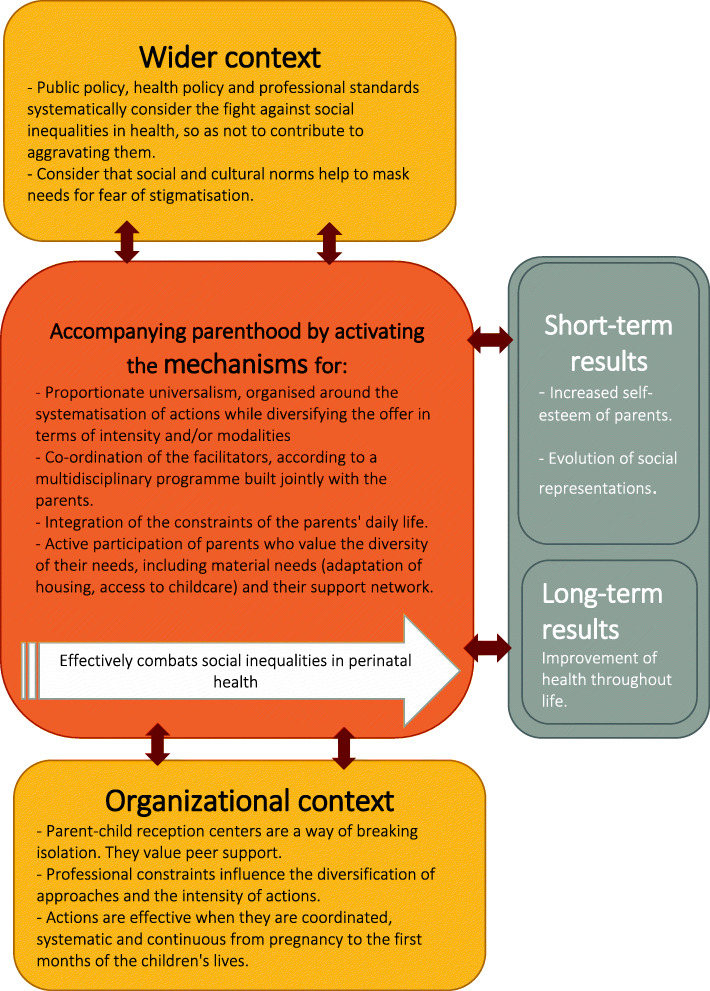
Middle-range theory of combatting inequality during the perinatal period

Certain context–mechanism–result configurations were found in the two neighboring territories studied. On the basis of recurring configurations, we present our refined, middle-range theory, as summarized in Fig. 3. The context elements, mechanisms, and short- and long-term results are presented in yellow, orange, and gray, respectively. The elements of the intermediate results are not shown in this diagram; however, they were described in the C–M–R configurations of the previous paragraph.

## Discussion

The main results concerning the fight against social inequalities in health show a true willingness on the part of those involved to carry out universal actions, coordinated between professionals and institutions, in response to the demands of parents; however, the reality on the ground shows the complexity of their implementation and the multiplicity of results. Overall, all the actions are aimed at improving the skills and knowledge of parents. These actions are collective (courses for parents, information sessions, and conferences) or individual around childcare issues. They take place in institutions (maternity wards and associations) or at home. The actions show little concern for fathers.

The results show that adapting to the needs of parents, described as an effective way of supporting parents, raises several questions highlighted by professionals. Parents who need help do not easily describe their difficulties due to a fear of:

- being stigmatized by showing their lack of self-confidence or knowledge, or their material distress,
- revealing their family lifestyle, which relates to the question of implicit norms expressed by professionals and social representations, and
- above all, the social services themselves. Indeed, the distinction between control and support seems to be critical to the reasons for non-access to the services offered by professionals.

The problem of professional organizations was also evoked: competition between disciplinary and sectoral fields, obstacles to communication, and cumbersome organizational and institutional procedures.

### Which levers can be used to combat social inequalities in health?

With regard to recommendations on the reduction of social inequalities in health, we now discuss the levers selected. Our middle-range theory showed that to be effective in tackling social inequalities in health, actions must address structural determinants at the macro-systemic level [1]. However, the field of realist evaluation shows that it is first and foremost the actions focused on individual behavior that are implemented.

Our theory shows us that proportionate universalism [38, 40, 53, 54] is potentially effective in reducing social inequalities in health. However, the populations that need support are those who do not ask for help; therefore, the gaps are widening as actions are more beneficial to parents who have a certain economic or educational affluence.

Integrating the diversity of families and the multiplicity of parental perspectives into the response provided by professionals is an effective approach. However, our observations teach us that the hierarchy of expert and lay knowledge is a hindrance to this adaptation to the needs of families.

While there is a general political desire to combat social inequalities in health in early childhood, these examples show that the strategies in place are potentially not the most effective. Effective support actions would respond to individual strategies; however, the current approaches target parents’ behavior, aiming to empower them but without giving them the means to do so. Thus, the conceptual evolutions of the 1980s and 1990s, which transformed the models explaining health status, have not been translated into operational health interventions and policies, with professional practices still marked by the biomedical model. More than 30 years after the Ottawa Charter [55], the shift from prevention to health promotion remains to be made [56].

### Parents: father and mother?

The evidence from the interviews is consistent with the findings of the literature review on the issue of parenthood, which mainly includes actions targeting only mothers. This issue of father–mother asymmetry was recently analyzed in a Swiss study [57], which confirmed that becoming a parent accentuates gender inequalities.

This issue is not specific to the scope of this work and relates to social representations, women’s level of education and educational backgrounds, the sharing of tasks within the family, the gender pay gap, access to and duration of parental leave, and professional practices related to support for parents.

### Support, assistance, or control?

The delicate borderline between support and control relates directly to issues of equity. First of all, the allocation of joint support and assessment missions for parents by the same service is a challenge. This relates to our hypothesis that parents who need such services most are the ones most likely not to use them, for fear of stigmatization or punishment.

Moreover, questions need to be raised about the reference frameworks on which the normative elements of good parenting and risk situations are based. How can we standardize the reference frameworks when the literature review points out that parenting is unique for everyone, and is not solely the responsibility of individual parents [5, 16]? Finally, since tasks relating to child protection are defined as priorities, they leave little room for universal measures.

These considerations are in line with the comments of Martin et al. in their report “Accompanying parents in their educational and care work”. These authors consider these issues with a view to identifying the role of public action: “Childcare workers […] who had mobilized a great deal of energy to deviate from the mission that had historically been entrusted to them, of monitoring and normalizing behavior, have been caught up in a new mission they have been asked to do, of assessing danger, particularly in response to any information that is worrying” [58].

### Learning to be a parent?

While the literature reviews which constituted our initial theoretical elements are for the most part centered on the practices of professionals, the programs studied essentially concern the improvement of mothers’ knowledge and capacities. Professional knowledge and skills are in line with the practices of risk assessment mentioned above.

However, focusing on individual behavior raises questions. Social epidemiology, life course epidemiology, and work in the social sciences have constantly demonstrated over the last few decades that individual behavior is influenced by social contextual elements that are far removed from that individual behavior. Interventions aimed at behavioral change are more likely to aggravate social inequalities in health than those that act on structural or political elements [59, 60]. By extension, questions can be raised, on the one hand, about inter- and multi-professional collaborations and, on the other hand, about the material needs of parents, such as access to adequate housing or access to suitable childcare. Adapting to the problems and demands of families, as diverse as they are numerous, runs counter to institutional standards and protocols. Professionals, therefore, seem to be trapped by contradictory injunctions.

Thus, knowledge and capacities underlie the hierarchical dimension of expert and lay knowledge. It should be remembered that most actions are carried out by professionals for the benefit of parents, raising the implicit question of a hierarchy of knowledge [5, 38, 61]. In this sense, traditional knowledge, group knowledge, and knowledge resulting from mother-to-daughter transmission tend to be devalued. However, it seems worthwhile recalling that childcare was born out of the desire for health control, based on a medical discourse that is both injunctive and normative [62]. Peer groups, in informal meeting places, not centered on educational activities, seem to be an avenue that professionals themselves value [2, 4, 6]. The isolation of mothers remains an important element to be considered in parallel.

### The support network: how to include it?

The literature review has shown that the relational aspect, the social bond, is little studied even though it represents a solid means of preventing or reducing the risks of depression, stress, anxiety, and other mental illnesses, and increases the feeling of control. However, this review of reviews reveals that studies focusing on listening skills and the quality of the professional-mother-parent-newborn relationship are under-represented. Even though the field survey showed that parents were largely supported by their family circle, the inclusion of these carers in action remains an open question.

### Associations, healthcare provision, and the private sphere

At a time when parents fear intrusions into the private sphere, when expert advice does not seem to be the only response to their needs that they expect, it would be interesting to question the raison d’être of associations in relation to parenting support. The range of services provided by associations complements the health services on offer in terms of content (workshops, conferences, and discussion groups), methods (multiple languages and long opening hours), and proposals (social cloakroom and information on self-help networks). Is this to make up for institutional shortcomings?

### Strengths and limitations

In the realist approach, it is the recurrence of observations through cases that allows the theory to be progressively refined. Two cases thus allow a first approach but do not form a basis for drawing definitive conclusions; further observations are necessary.

We used quantitative data to consolidate the data from our qualitative research, and vice versa. Diversifying our data sources means that the quantitative data allow for an explanatory approach that complements the qualitative data by taking socio-epidemiological factors into account. The diversity of the data serves to limit the subjectivity associated with one method or the other.

It should be noted that, despite the respective limitations of the previously published literature review [1] and this case study, it appears that they are consistent in their conclusions; this reinforces the validity of our results.

## Conclusion

This research constitutes a body of knowledge gathered for reflection and action. In Luxembourg, the Directorate of Health is currently carrying out a large survey (the European Health Interview Survey) [63] to gain a better understanding of the current state of health of their residents, with the primary objective of adapting the health system to the real needs of citizens.

The Haut Conseil de Santé Publique [64] recommended that France should clearly state the objective of reducing social inequalities in health in its programs and actions. Our conclusions provide some ideas on how to operationalize this recommendation. In particular, any perinatal policy should clearly state among its objectives the intention to reduce social inequalities in health. The policy should also state that it will be evaluated according to the criteria of proportionate universalism, interprofessional coordination, and actions based on the diversity of parents’ needs. In France, these recommendations are in line with the recent proposals of the experts of the First 1000 Days Commission, in particular the creation of personalized support from pregnancy to the post-partum return home, the strengthening of professional partnerships between different institutions, and the extension of paternity leave [65]. Our results could contribute to the effective implementation of this ambitious plan.

## Supplementary Information

**Additional file 1.** Interactions between socio-ecological systems applied to parenting in the perinatal period.

**Additional file 2.** Focus group discussion guide.

**Additional file 3.** English language questionnaire.

**Additional file 4.** Framework for interpreting the documentary material based on the REFLEX-ISS tool.

**Additional file 5.** Synthesis of the research design regarding parenting support interventions against social inequalities in health.

## Data Availability

Data from the survey are available on request to Annabelle Pierron, corresponding author.

## Abbreviations

ATIH: Agence Technique de l’Information sur l’Hospitalisation
C: Context (in the configuration context-mechanism-Result)
COREQ: COnsolidated criteria for REporting Qualitative research
CNAM: Caisse Nationale d’Assurance Maladie
CNER: Comité National d’Ethique de la Recherche
CNS: Caisse Nationale de Santé
DREES: Direction de la Recherche des Etudes de l’Evaluation et des Statistiques
FR: FRance
INSEE: Institut National de Statistique de d’Etudes Economiques
LU: LUxembourg
M: Mechanism (in the configuration context-mechanism-result)
MRC: Medical Research Council
PMI: Protection Maternelle et Infantile
R: R (results in the configuration context-mechanism-outcome)
RAMESES: Realist And Meta-narrative Evidence Syntheses: Evolving Standards
STATEC: institut national de la STATistique et des études EConomiques
WHO: World Health Organization

## Footnotes

1 Terms and conditions can be consulted at https://guichet.public.lu/fr.html for Luxembourg and https://www.service-public.fr for France.
