# Supplementary material for: Supporting parents by combatting social inequalities in health: a realist evaluation
Source: BMC Public Health. 2021 Jun 29;21:1252. doi: 10.1186/s12889-021-11237-2 (PMC8244179; doi:10.1186/s12889-021-11237-2)
Supplement: Supplementary file 2 — Additional file 2. Focus group discussion guide. [file 12889_2021_11237_MOESM2_ESM.docx]

**Parenthood and health promotion for mothers and newborns**

**Assessment in relation to social inequalities in health**

Qualitative interview guide for professionals in the medico-psycho-social and associative sectors.

Explanatory note:

This guide lists the themes to be addressed in the interview and a series of questions to be adapted: to the context (structure: association, hospital, private practice), and to the profile of the interviewee (voluntary association, private practice or hospital activity).

The aim is to gather in-depth information on the experiences of certain key players. The comparison of the different actors' discourses, the parents' self-questionnaires and the documentary data will enable the development and implementation process to be modelled from an evaluative perspective.

The focus groups will be the object of observations and note-taking; this will be followed by a transcription and then an analysis with the aim of theorising the interactions at the level of the different elements of the ecological model developed initially (inspired by the systems theory model).

The verbatims of the individual interviews will be analysed for content and processed by the Nvivo® software.

Procedures for contacting professionals :

1/ Presentation of the project to the various directors of establishments, network coordinators, heads of department, team leaders.

2/ Focus groups of the actors concerned during multidisciplinary meetings, interdisciplinary if necessary.

3/ Individual interviews with key professionals involved.

**Presentation of the study**

*Hello,*

*This study - carried out by the University of Lorraine in partnership with the Luxembourg Institute of Health - aims to understand how intervention projects relating to support for parenthood were developed and how they were implemented. We are interested in different structures (hospital, associations) and different actors (health and psycho-social professionals) involved in order to cross the points of view and the practices.*

*The questions I am going to ask you therefore concern your personal experience, your vision of things, and more particularly the obstacles but also the levers that could be considered. There is no right or wrong answer, only your experience.*

*I will take notes during the interview and, if you agree, I will record our discussion. This recording will only be listened to by me in order to complete my notes and it will be destroyed at the end of the study. It will therefore remain anonymous and confidential. Your answers will be included anonymously in the final report of this study.*

*The interview should last approximately one hour.*

#### Presentation of the interviewee

1. Could you tell me about your current position and your professional background? How long have you been working in this structure/as a private practitioner?

2. How would you define your function in relation to the support of parents?

- Do you consider that you are a mediator of this support towards other institutions, actors, professionals?

- Do you consider that you have a role in promoting health, in health education through this support?

- Personally, what was your objective in getting involved in the health/support of mothers, couples and parents?

- Are you specifically paid for these interventions?

- Does this represent the largest part of your activity?

#### Development and implementation of interventions/actions/sessions with parents

3. Do you have any general knowledge of the means currently in place to support and accompany parents during the period from pregnancy to the first months of the child's life (until the end of maternity leave)?

- At local/departmental/regional/national level?

- Who (actors or institutions) support the approach? Who seems to be the most mobilised?

- On the contrary, which actors seem to you to be more reluctant/more difficult to mobilise?

- Are you aware of the way in which these projects have been implemented?

- Do you know if certain actors were "added" during the development/implementation? which actors "disappeared"/went into the background?

4. Why did your institution/association/structure want to be involved in supporting parents, especially during the period around the birth?

5. Could you present the interventions/actions/sessions and the objectives/themes?

- What objectives were defined first? Did you determine these objectives yourself or was it the result of a group work?

- Were the themes addressed defined according to the parents' requests? how did you identify these requests?

- Are the issues addressed different according to the groups they are aimed at: women, men, first child, usual language spoken, level of education, material conditions of families, social conditions of families?

- Which objectives were defined later?

- Which themes were debated?

- Which themes were abandoned?

6. Have you ever participated in multidisciplinary meetings related to parenting support?

- Who were the actors present?

- What were the topics of discussion?

- What difficulties were anticipated/ discussed?

- What tools (in a broad sense) have been developed?

7. What were the tools/procedures/partnerships that facilitated the setting up and/or running of the sessions/actions/interventions?

- On the contrary, what did you miss (in the broadest sense of the term)?

- What difficulties/constraints did you (have) encountered?

- Which of these constraints have been overcome? How were they overcome?

- What helped you? (tools, procedures, actors...)?

#### Partnerships and coordination

#### 8. With which actors or institutions do you have contact in the framework of these sessions/actions/interventions related to parenting support?

#### - Could you describe your relationships?

#### - How do you communicate? How often?

#### (list all the actors and institutions in order to be able to "draw" a sociogram)

#### 9. Are you aware of any coordination of projects relating to support for parenthood, how has the involvement of the various actors evolved?

#### - How are the meetings between the actors fixed?

#### - Who centralises the information?

#### - Are there any tools on which this coordination is based?

#### Perceived effects/social inequalities in health / epidemiology

10. Since you have been taking part in - or orienting yourself towards - these actions/interventions/sessions, do you perceive any changes?

- in the relations between actors/professionals/associations?

- within your institution/structure?

- in funding?

- in the working methods?

- in the partnerships?

11. In your opinion, what are the elements that show the impact of these interventions on the health of mothers, fathers, couples and newborns?

- What evidence would you have to believe/confirm this?

- Do you think that the impact is the same whatever the audience (single mother, couple, expecting a first child or parent of a family already formed)

12. In your opinion, what are the main difficulties encountered that could hinder/limit the efforts undertaken/achieve different objectives?

- Do you think you are reaching the public that most needs to be supported?

- What are the most difficult actions to implement? Why or why not? What means could facilitate their implementation?

- What are the most difficult objectives to achieve? Why or why not?

#### Assessment

13. Finally, which actors do you consider to be key in the actions/sessions support for parenthood.

- Which partners do you consider to be key but absent/not present enough? Do you know the reasons for this absence?

14. In your opinion, which actors/institutions were the most involved?

- How did this involvement translate? in terms of initiative, in terms of means, financial, human...

15. What do you think should be changed/improved/deleted?
