# Supplementary material for: Supporting parents by combatting social inequalities in health: a realist evaluation
Source: BMC Public Health. 2021 Jun 29;21:1252. doi: 10.1186/s12889-021-11237-2 (PMC8244179; doi:10.1186/s12889-021-11237-2)
Supplement: Supplementary file 3 — Additional file 3. English language questionnaire. [file 12889_2021_11237_MOESM3_ESM.doc]

/ / /

**Survey to improve for a best supporting mothers, fathers and newborns**

**Survey presentation**

This survey is about a public health research. With this questionnaire, it is about understanding the different possibilities available to you to accompany your pregnancy, during the pregnancy of your spouse, and during the first months with a newborn baby.

To answer this questionnaire, you need 15 to 20 minutes.

**Fill Guide**
The questionnaire is **anonymous**. It includes predefined themes and questions, but it also aims to adapt to each case:

The first part defines your family, professional and economic conditions.

The second part addresses the question of the support methods offered to parents.

The questionnaire can be completed directly on this paper or as part of a telephone interview with the research team. To make an appointment, you can contact us: annabelle.pierron@univ-lorraine.fr

**Analysis of the questionnaire**

The questionnaires will be analyzed by the research team, in accordance with the rules of confidentiality. Each of the answers will be coded via the Epidata® software, then a content analysis will be done via the NVivo software. The results of this survey will be the subject of public communication and will contribute to the development of a publication in a peer-reviewed scientific journal.

**You can mail the questionnaire using the enclosed envelope. You can also drop it here.**

**We thank you for your participation.**

**Let's talk about your life context**

**Q1. What is your gender?**

- Women
- Men

**Q2a. Are you pregnant, or your spouse is pregnant?**

- Yes. You or your spouse is pregnant : how many months? ………………………….
- No, your baby is borned. How old is the baby?.......................................................

**Q2b. Are you (your spouse) pregnant with twins or yours twins are borned?**

- Yes
- No

**Q3. How old are you ?**...........................................................................................................................

**Q4. How many children have you?** ……………………………………………………………………….

**Q5. How old are they?**

First child : ………………………

Second child : ………………………

Third chid : ……………………….

Fourth child : ……………………….

Fifth child : ……………………….

**Q6. Do you live with your spouse?**

- yes
- no. *You can go to Q19a*

**Q7. How old are your spouse?**.......................................................................................

**Q8. How many child have you with your spouse?**

- ………..
- None

**Q9. You or your spouse, did you have children from a previous couple?**

- No
- Yes, your spouse
- Yes, you
- Yes, you both

**Q10. Do you consider that your spouse is interested in your personal or professional life?**

- Yes, he, she is interested
- No, he, she does not care

**Q11.** I**n your home, how is the division of household chores distributed ?**

- You realize the totality
- You achieve significantly more than half
- You make about half
- You achieve significantly less than half

**Q12. For domestic tasks, you are helped by**:

- Your spouse
- By a third person, can you specify
- You are not helped

**Q13.** **For the custody of your child (ren), you are (will be) helped by**:

- Day nursery
- Your family
- A nursery assistant, a childminder
- Other, could you precify: ……………………………………………………………………………
- You are (will) not helped

**Q14. Would you say that in your couple you are autonomous for the education of your child (children), for example concerning the decision making, the daily life of your child(ren)?**

- Yes, absolutely
- Oui, rather
- No, not really
- No, not at all

**Q15. Tell your partner that you are supportive of the care that will be provided to your child (s) (changes, sleep, eating) ?**

- Yes, absolutely
- Oui, rather
- No, not really
- No, not at all

**Q16. According to you during the period following maternity leave, what is the best?**

- The mother stops working to take care of it
- The father stops working to take care of it
- One of the parents, indifferently, stops working to take care it
- It is not essential for parents to stop working

**Q17. Do you and your spouse have the same vision regarding the care of your child (ren) or the education of your child (ren)?**

- Yes, exactly
- Tes, nearly
- No. There are small differences between us.

Which :………………………………………………………………………………………………

- No. There are big differences between us..

which :…………………………………………………………………………………………….....

**Q18. If No: Do you think these differences relate to the education you received as a child?**

*Please circle the answer that best suits you :*

**Q19a. Do you currently have a professional activity?**

- Yes
- No. *Go to Q21.*

**Q19b. As a parent or future parent, have you ever considered reducing your professional activity?**

- Yes and I have reduced my professional activity
- Yes and I haven’t reduces my professional activity
- No
- You haven’t a profesionnal activity

**Q19c. Are you border?**

- Yes
- No

**Q20. Are you currently on parental leave, or do you plan to take parental leave ?**

- Yes. How many time?......................................................................................
- No

**Q21. Is your spouse currently on parental leave or does he (she) intend to take parental leave?**

- Yes
- No
- You do not have a spouse

**Q22. Are you satisfied with the time you can devote to your** **child(ren)?** *Please circle the answer that best suits you:*

**Q23. Are you satisfied with the time you can devote to your couple ?**

**Q24. Are you satisfied with the time you can spend with your other relatives (friends, relatives, neighbors ...)?**

**Q25. During your pregnancy (or the pregnancy of your spouse) have you (she) been hospitalized?**

- Yes. can you specify the number of hospitalizations greater than one night ?..................
- No

**Q26. During your last pregnancy (or current pregnancy), how long have you been on sick leave ?**

Number of weeks: ……………………………………………………………………………………..

**Q27. Have you (or your spouse) breastfed your last child or are you planning to breastfeed (plans to) ?**

- Yes. Could you explain why : ………………………………………………………………………..
- No. Could you explain why : ………………………………………………………………………..

*For women and if you have already given birth, please continue Q28.
Future mother, future father : you can continue Q38.*

# Concerning chidbirth

**Q28a. Where did you give birth for your last child ?**

- At the maternity hospital of Esch-sur-Alzette
- Ay the maternity hospital of Mont-Saint-Martin
- In another maternity hospital in Luxembourg. Which :………………………………………….
- In other maternity hospital in France. Which :…………………………………………………….
- At home

**Q28b. How was your last birthchild ?**

- Your delivery has been provoked (induced labor) ❒ Oui ❒ Non
- You gave birth naturally ❒ Oui ❒ Non
- You have delivery at full term ❒ Oui ❒ Non
- Can you specify which term : ………………………………………………………………………

**Q29. What was the weight of your last child at birth? …………………………………………………..**

**Q30. Was your last child hospitalized?**

❒ Yes ❒ No

Can you specify:  ………………………………………………………………………………………

**Q31.Your child (children) is (are they all) healthy?**

❒ Yes ❒ No : can you specify?..............................................................................................

**Q32. How many days did you spend at the maternity ward after giving birth ?**

……………………………………………………………………………………………………………..

**Q33. Are you satisfied with your maternity stay in terms of duration ?**

*Please circle the answer that best suits you :*

**Q34. Are you satisfied with your maternity stay in terms of the availability of professionals ?**

**Q35. Are you satisfied with your maternity stay in terms of advice and information provided?**

**Q36. Are you satisfied with your maternity stay in terms of adapting to your requests?**

**Q37. Are you satisfied with your stay at the maternity ward in terms of the number of visits by your loved ones?**

# From a material point of vue

**Q38. Do your financial means allow to meet your needs (your health and the health of your child (ren), childcare, transport ...) ?**

- Totaly
- Mostly
- In the minority
- Not at all

**Q39. Do you ever renounce certain expenses because you have not enough money?**

- Yes, including for essential expenses (food, health, housing)
- Yes, but only for optional expenses (leisure for example)
- No

**Q40. Does the size of your home seem adapted to your needs?**

**Q41. Does the equipment (eg furniture, appliances, heating) and the layout of your home seem appropriate to your needs?**

# About your entourage

**Q42. Generally, from whom do you find support ?** *many possible responses*

- Your spouse ❒ Yes ❒ No
- (a) colleague(s) ❒ Yes ❒ No
- (a)friend(s) ❒ Yes ❒ No
- A member of your family ❒ Yes ❒ No. Can you specify:………………………
- A neighbor ❒ Yes ❒ No
- Somebody else ❒ Yes ❒ No. Can you specify:………………………

**Q43. Have you ever felt the need to speak to a professional to be reassured ?**

- Yes, and I asked for one. Can you be more precise……………………………………………
- Yes, but I finally did not ask
- No

If yes, can you clarify this problem that led you to speak to a professional …………………………………………………………………………………………………………………………………………………………………………………………………………………………

**Q44. Regarding the pregnancy, to which type of professional do you go (did you go) when you have a doubt, a question, an uncertainty?**

- A health professional **in PMI, or Ligue Medico-Sociale** (doctor, midwife, nurse…), can you specify……………………………………………………………………………………………..
- A health professional **in city or hospital consultation,** can you specify……………………..
- A professional in education, can you specify…………………………………………………….
- A professional from the social sector, can you specify…………………………………………
- A professional practicing within an association, can you specify……………………………
- Another professional, can you specify…………………………………………………………….

**Q45. Concerning childbirth, to which type of professional do you go when you have (had) a doubt, a question, an uncertainty?**

- A health professional **in PMI, or Ligue Medico-Sociale** (doctor, midwife, nurse…), can you specify……………………………………………………………………………………………..
- A health professional **in city or hospital consultation**, can you specify……………………..
- A professional in education, can you specify…………………………………………………….
- A professional from the social sector, can you specify…………………………………………
- A professional practicing within an association, can you specify……………………………
- Another professional, can you specify…………………………………………………………….

**Q46. Regarding the care of your child (ren), to which type of professional do you go when you have (had) a doubt, a question, an uncertainty?**

- A health professional **in PMI, or Ligue Medico-Sociale** (doctor, midwife, nurse…), can you specify……………………………………………………………………………………………..
- A health professional **in city or hospital consultation**, can you specify……………………..
- A professional in education, can you specify…………………………………………………….
- A professional from the social sector, can you specify…………………………………………
- A professional practicing within an association, can you specify……………………………
- Another professional, can you specify…………………………………………………………….

**Q47. Regarding the behavior of your child (ren), towards which type of professional do you go when you have (had) a doubt, a question, an uncertainty?**

- A health professional **in PMI, or Ligue Medico-Sociale** (doctor, midwife, nurse…), can you specify……………………………………………………………………………………………..
- A health professional **in city or hospital consultation**, can you specify……………………..
- A professional in education, can you specify…………………………………………………….
- A professional from the social sector, can you specify…………………………………………
- A professional practicing within an association, can you specify……………………………
- Another professional, can you specify…………………………………………………………….

**Q48a. From your point of view, did the pregnancy monitoring consultations reassure you?**

**Q48b. From your point of view, have you been reassured by the pediatric follow-up consultations?**

**Q49. In case of a serious problem (urgent or not), do you consider that you are sufficiently surrounded to face the situation?**

**Q50. Currently, how worried are you about the following topics?**

**Q50a. The pregnancy:**

❒ very worried ❒ a little worried ❒ not at all worried

**Q50b. The childbirth :**

❒ very worried ❒ a little worried ❒ not at all worried

**Q50c. Your youngest child :**

❒ very worried ❒ a little worried ❒ not at all worried

**Q50d. Your biggest child(ren) :**

❒ very worried ❒ a little worried ❒ not at all worried

can you specify :………………………………………………………………………………………

**Q50e. Life as a couple :**

❒ very worried ❒ a little worried ❒ not at all worried

**Q50f. Your job :**

❒ very worried ❒ a little worried ❒ not at all worried

**Q50g.Your health :**

❒ very worried ❒ a little worried ❒ not at all worried

**Q50h. Do you have other concerns ?**

Can you specify : ………………………………………………………………………………………

# Regarding information available to parents

**Q51. During pregnancy, do you feel that you are sufficiently informed about the progress of the pregnancy?**

❒ Yes, enough

❒ Yes, but not adequately

❒ No

**Q52. During pregnancy, do you feel that you are sufficiently informed about the progress of the childbirth ?**

❒ Yes, enough

❒ Yes, but not adequately

❒ No

**Q53. During pregnancy, do you feel that you are sufficiently informed about the progress of the behavior of a newborn ?**

❒ Yes, enough

❒ Yes, but not adequately

❒ No

**Q54. During pregnancy, do you have the feeling of being sufficiently informed about the professionals who can inform you, reassure you ?**

❒ Yes, enough

❒ Yes, but not adequately

❒ No

**Q55. During pregnancy, did you feel that you were sufficiently informed about the associations that accompany new-born parents?**

❒ Yes, enough

❒ Yes, but not adequately

❒ No

**Q56. The last time you needed information about pregnancy, childbirth, or daily newborn, where did you go first?**

- A book or a magazine
- Internet
- TV or radio
- A professionnal. Which one : …………………………………………………………………
- A relative. Which one : ………………………………………………………………………..

# Concerning professionnals who accompany parents

**Q57. Do you have, near your home, a place to welcome or help families, for parents?**

- Yes
- No
- You don’t know

**Q58. Have you ever heard of a parent-child “home “?**

- *For example in France: Early childhood center of Mont-St-Martin, Longwy town hall LAPE, Longwy toy library, center of PMI Maternal and Infant protection in Longwy.*
- *For example in Luxembourg: the parents 'house Elternhaus in Esch-sur-Alzette, the parents' cafe in Esch-sur-Alzette, the Initiativ Liewensufank in Itzig, baby-plus service in Dudelange, Leche League Esch-sur-Alzette.*
- Yes. How have you been informed?........................................................................................
- No

**Q59. Did you use the services offered by the parent-child “home”?**

- Yes, several times
- Yes, only once
- No, never

If yes, for which service? …………………………………………………………………………….

**Q60. Have you ever heard of "birth preparation" sessions?**

*For example: at CHEM maternity ward, Mont-Saint-Martin maternity ward, Briey maternity ward, with a liberal midwife, with a psychologist, with a professional practicing haptonomy, yoga ...)*

- Yes. How were you informed ? ……………………………………………………………………
- No. *Go to Q70*

**Q61. Did you participate in these sessions?**

- Yes, several times
- Yes, only once
- No, never. *Go to Q70*

If yes, for which motive ?……………………………………………………………………………

**Q62. Have you been satisfied with the length of the "preparation for birth" sessions?**

**Q63. Have you been satisfied with the schedules?**

**Q64.** **Were you satisfied with the location of these sessions?**

**Q65. Were you satisfied with the speaker who facilitated these sessions ?**

**Q66. Have you been satisfied with the program of these sessions ?**

**Q67. Have you been satisfied with the language used by the speaker ?**

**Q68. Were these sessions tailored to your needs ?**

**Q69. Would you say that these sessions helped you?**

**Q70. Have you ever heard of consultations or speaking groups related to breastfeeding or parenting?**

- Yes. How were you informed ? ………………………………………………
- No. *Go to Q80*

**Q71. Did you participate in these consultations or talk group ?**

- Yes, several times
- Yes, only once
- No, never. *Go to Q80*

If yes, for which raison? ……………………………………………………………………………

**Q72. Have you been satisfied with the length of consultations or talk group related to breastfeeding or parenting?**

**Q73. Have you been satisfied with the schedules ?**

**Q74. Have you been satisfied with the place ?**

**Q75. Were you satisfied with the speaker ?**

**Q76. Have you been satisfied with the content, program ?**

**Q77. Have you been satisfied with the language used by the speaker ?**

**Q78. Were these consultations or group of words adapted to your needs?**

**Q79. Did these consultations or talk groups help you?**

**Q80. Have you ever heard of home visiting for parents ?**

*For example: visit of a midwife, nursery nurse, professional liberal or Maternal and Infant Protection or ALUPSE baby?.*

- Yes. How were you informed ? ……………………………………………………………………...
- No. *Go to Q90*

**Q81. Did you use these home visits ?**

- Yes, several times
- Yes, only once
- No, never. *Go to Q90*

If yes, for which raison? ………………………………………………………………………………

**Q82. Have you been satisfied with the duration of these home visits ?**

**Q83. Have you been satisfied with the schedules of these home visits?**

**Q84. Have you been satisfied with the number of visits ?**

**Q85. Have you been satisfied with the professional?**

**Q86. Have you been satisfied with the issues addressed during these visits (eg advice, information)?**

**Q87. Have you been satisfied with the language used by the speaker ?**

**Q88. Were these home visits tailored to your needs ?**

**Q89. Would you say that these home visits helped you?**

# Let's finish with your portrait

**Q90. In which city do you live ? ……………………………………………………………………………...**

**Q91. Where was your last child born / in which city is it planned for you (or your spouse) to give birth (give birth)**?

- At the maternity hospital of Esch-sur-Alzette
- At the maternity of Mont-Saint-Martin
- An other luxembourg maternity. Which one ?…………………………………………………..
- An other french maternity. Which one ?………………………………………………………….
- At home

**Q92. What is your nationality ?**...............................................................................................................

**Q93. What is your marital status with your spouse ?**

- Youe are in concubinage
- You are in «  pacs » . in which year : ………………………………………………………….
- You are married. In which year : ………………………………………………………………
- You haven’t spouse

**Q94. At what age did you finish your studies?** ……………………………………………………………

**Q95. What is your highest degree ?**

- No diploma
- Brevet, BEP, CAP, vocational or technical training (below baccalauréat level)
- baccalauréat
- Higher technical training as BTS,DUT, BAC +2, BAC+3, bachelor
- University education, engineering education, master, Bac +5 and more

**Q96. What is your current job situation ?**

- You work

How many hours for one week do you work ?………………………………………………..

- You have no paid professional activity
- You are looking for a job

**Q97. What is exactly your current job situation?**

- You have a permanent contract or are an official
- You have a fixed term contract
- You are independent, to your account
- You have another type of job

**Q98. What is the socioprofessional category closest to yours ?**

- Worker
- Employee
- Middle manager, technician, teacher, nurse…
- Senior Manager, Engineer, Teacher
- other: …………………………………………………………………………………………………

**Q99. Do you receive social benefits (family allowances, social housing assistance ...)**

- Yes , what is the monthly amount? ……………………………………………………………
- No

**Q100. How are your personal income divided?**

- Your income is mainly from your professional activity
- Your income comes mainly from help (from my relatives or social assistance)
- Other :…………………………………………………………………………………………………….
- You haven’t personal income

**Q101. What is the monthly amount of your personal net income?**

- Less than 999 euros
- 1000 to 1999 euros
- 2000 to 2999 euros
- 3000 to 3999 euros
- 4000 to 4999 euros
- 5000 to 5999 euros
- More than 6000 euros

**Q102. What is the monthly amount of your household's overall net income ?**

- Less than 999 euros
- 1000 to 1999 euros
- 2000 to 2999 euros
- 3000 to 3999 euros
- 4000 to 4999 euros
- 5000 to 5999 euros
- More than 6000 euros

**Regarding your spouse**

**Q103. At what age did he finish his/her studies?...............................................................................**

**Q104. What is his/her highest degree ?**

- No diploma
- Brevet, BEP, CAP, vocational or technical training (below baccalauréat level)
- baccalauréat
- Higher technical training as BTS,DUT, BAC +2, BAC+3, bachelor
- University education, engineering education, master, Bac +5 and more
- You don’t know

**Q105. What is his/her current job situation ?**

- She, he works

How many hours for one week do you work ?………………………………………………..

- She, he has no paid professional activity
- She, he ‘s looking for a job

**Q106. What is exactly his/her current job situation?**

- She, he is a permanent contract or are an official
- She, he is have a fixed term contract
- She, he is independent, to your account
- She, he has another type of job

**Q107. What is the socioprofessional category closest to him/her ?**

- Worker
- Employee
- Middle manager, technician, teacher, nurse…
- Senior Manager, Engineer, Teacher
- other:: ………………………………………………………………………………………………….

**Even if your father, mother is deceased, unemployed, or retired, try to answer**

**Q108. What is you father’s highest degree ?**

- No diploma
- Brevet, BEP, CAP, vocational or technical training (below baccalauréat level)
- baccalauréat
- Higher technical training as BTS,DUT, BAC +2, BAC+3, bachelor
- University education, engineering education, master, Bac +5 and more
- You don’t know

**Q109. What is you mother’s highest degree ?**

- No diploma
- Brevet, BEP, CAP, vocational or technical training (below baccalauréat level)
- baccalauréat
- Higher technical training as BTS,DUT, BAC +2, BAC+3, bachelor
- University education, engineering education, master, Bac +5 and more
- You don’t know

**Q110. In which place has this questionnaire been made available to you?**

- In a private practice. Please circle the answer:
  pediatrician. gynecologist. general practitioner. midwife. psychologist. physiotherapist
- Within an association
- At the PMI, at the medico-social league SPSE
- With the social worker
- At the maternity ward
- In an other place

**Thank you for completing this questionnaire.**
