# Supplementary material for: Supporting parents by combatting social inequalities in health: a realist evaluation
Source: BMC Public Health. 2021 Jun 29;21:1252. doi: 10.1186/s12889-021-11237-2 (PMC8244179; doi:10.1186/s12889-021-11237-2)
Supplement: Supplementary file 4 — Additional file 4. Framework for interpreting the documentary material based on the REFLEX-ISS tool. [file 12889_2021_11237_MOESM4_ESM.docx]

**Framework for interpreting the documentary material based on the REFLEX-ISS tool.**

| **Areas** | **Elements for documentary analysis** |
| --- | --- |
| **Type of document** | Website, flyer. Location. Method of provision (posted advertisement, document given out, through a third party) adapted to the fight against social inequalities of health. Target subgroups. |
| **Planning** | Type of target subgroups, social determinants of health targeted by the intervention. Sources of information referenced. Framing the project objectives in terms of an action plan to address social determinants of health. Expressing commitment to involving target subgroups and stakeholders. |
| **Implementation** | Type of intervention. Work methods to encourage the participation of target subgroups and stakeholders. Defining roles, tasks, and responsibilities. Sharing leadership. Supporting the acquisition of knowledge and competencies. Adapting to the literacy level of the target subgroups. |
| **Evaluation*** | Integration and participation in the evaluation. Long-term evaluation and undesirable outcomes. |
| **Sustainability*** | Activities to ensure intervention results are sustainable. Putting in place human, organizational, and financial resources to support the intervention in the long-term. |
| **Empowerment** | Activities aimed at developing self-esteem, critical awareness, competencies, and participation of target subgroups and stakeholders. |

Framework for interpreting the documentary material based on the REFLEX-ISS tool

(*) : Where the document allowed us to analyze this
