# Supplementary material for: Supporting parents by combatting social inequalities in health: a realist evaluation
Source: BMC Public Health. 2021 Jun 29;21:1252. doi: 10.1186/s12889-021-11237-2 (PMC8244179; doi:10.1186/s12889-021-11237-2)

## **Synthesis of the research design regarding parenting support interventions to fight against social inequalities in health.**


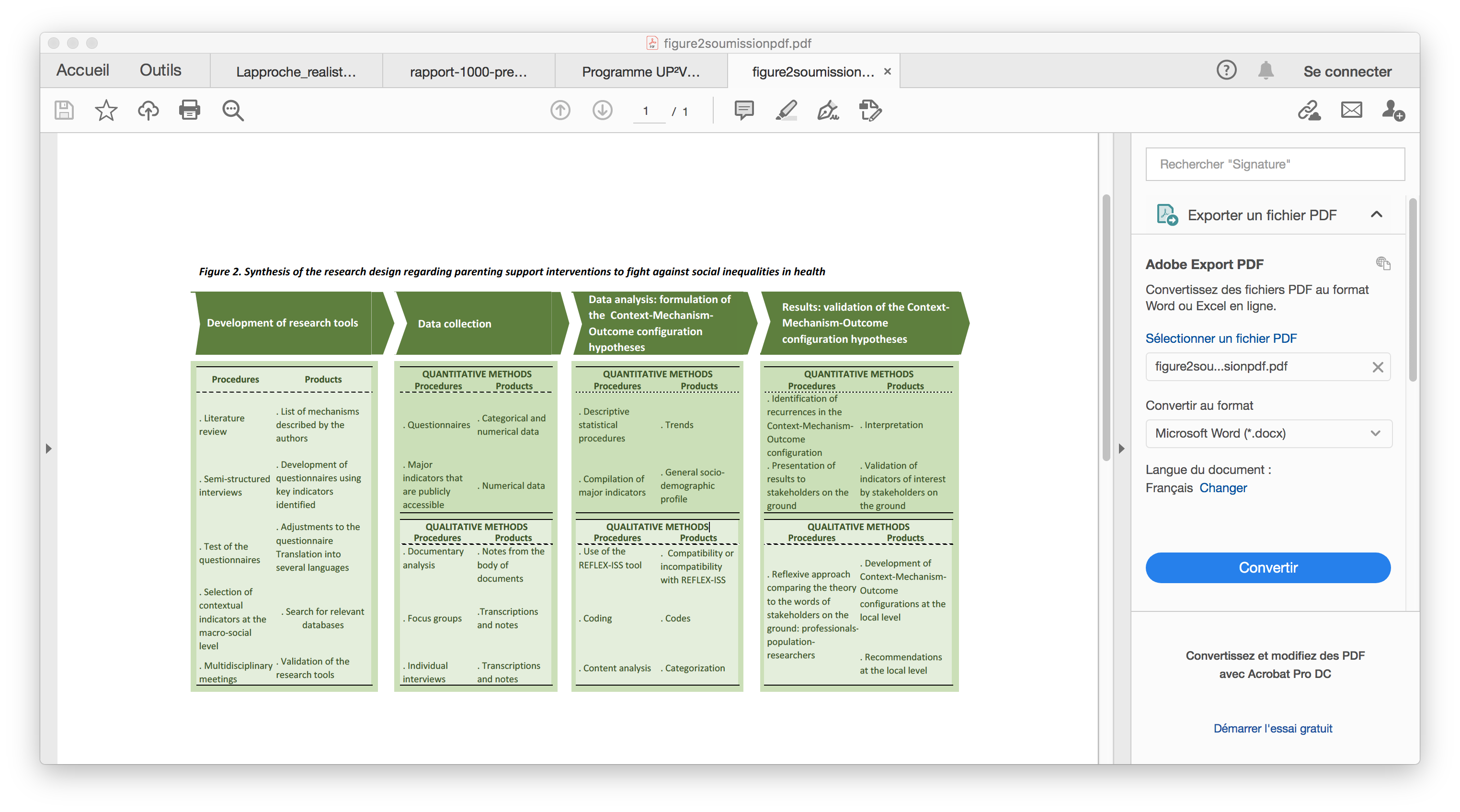

Supplement: Supplementary file 5 — Additional file 5. Synthesis of the research design regarding parenting support interventions against social inequalities in health. [file 12889_2021_11237_MOESM5_ESM.docx]
