# Supplementary material for: Supporting parents by combatting social inequalities in health: a realist evaluation
Source: BMC Public Health. 2021 Jun 29;21:1252. doi: 10.1186/s12889-021-11237-2 (PMC8244179; doi:10.1186/s12889-021-11237-2)
Supplement: Supplementary file 5 [file 12889_2021_11237_MOESM1_ESM.docx]

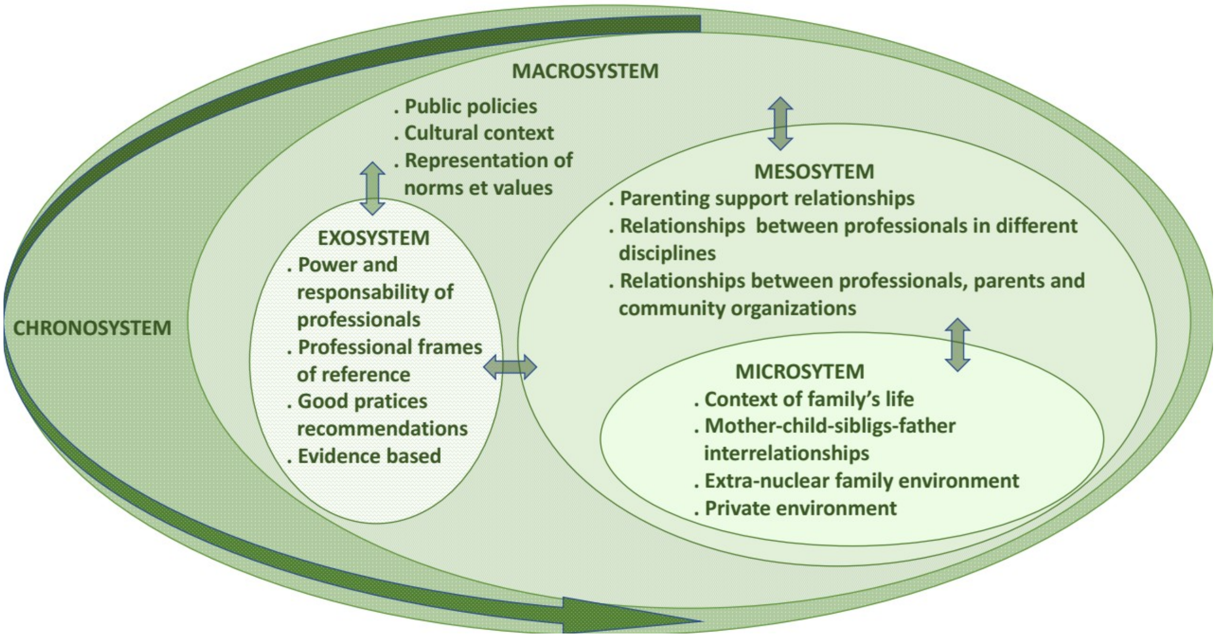
MACROSYSTEM
. Public policies
. Cultural context
. Representation of norms et values
MESOSYTEM
. Parenting support relationships
. Relationships between professionals in different disciplines
. Relationships between professionals, parents and community organizations
EXOSYSTEM
. Power and responsability of professionals
. Professional frames of reference
. Good pratices recommendations
. Evidence based
CHRONOSYSTEM
MICROSYTEM
. Context of family's life
. Mother-child-sibligs-father interrelationships
. Extra-nuclear family environment
. Private environment
